# The Relationship Between School Disconnectedness and Adolescent Internet Addiction: A Moderated Mediation Model

**DOI:** 10.3390/bs16010101

**Published:** 2026-01-12

**Authors:** Gonglu Cheng, Xuejun Bai, Shuying Fu

**Affiliations:** 1 Key Research Base of Humanities and Social Sciences of the Ministry of Education, Academy of Psychology and Behavior, Tianjin Normal University, Tianjin 300387, China; 2 Faculty of Psychology, Tianjin Normal University, Tianjin 300387, China; 3 Jiangsu Provincial University Key Lab of Child Cognitive Development and Mental Health, Yancheng Teachers University, Yancheng 224002, China

**Keywords:** school disconnectedness, relative deprivation, adversity beliefs, internet addiction

## Abstract

Internet addiction (IA) refers to an individual’s excessive and uncontrollable internet use. According to the cognitive-behavioral model of IA, school disconnectedness and relative deprivation may function as environmental and cognitive risk factors for adolescent IA, while adversity beliefs could serve as a protective factor. To examine whether relative deprivation mediates the link between school disconnectedness and adolescent IA, and whether adversity beliefs moderate this mediation, 2485 Chinese adolescents completed anonymous measures assessing school disconnectedness, relative deprivation, adversity beliefs, and IA. Results showed that: (1) school disconnectedness positively predicted IA; (2) relative deprivation partially mediated this relationship; (3) adversity beliefs moderated the relationship between school disconnectedness and IA; (4) adversity beliefs also moderated the link between relative deprivation and IA. These findings show the mediating role of relative deprivation and the moderating role of adversity beliefs in the association between school disconnectedness and IA, suggesting that fostering adversity beliefs could help reduce IA among adolescents.

## 1. Introduction

Internet addiction (IA) refers to an individual’s excessive and uncontrollable internet use (Kraut et al., 1998). With widespread internet adoption and the emergence of various types of online applications, IA has become a serious public health concern among adolescents.

Recent research estimates that the prevalence of IA among Chinese adolescents was 10.3% (Zheng et al., 2025). Adolescence is a period of developmental transition from childhood to adulthood, and their neural self-control systems are still developing. This makes them more sensitive to immediate rewards and external temptations (Willoughby et al., 2014). As a result, highly stimulating online content can be hard to resist, which can raise the risk of IA (Paus et al., 2008).

Evidence indicates IA is associated with multiple adverse outcomes, including physical health issues such as headaches and sleep disturbances (Demirer & Guenes, 2025; Krishnan & Chew, 2024), psychological problems such as depression and anxiety (Gull & Sravani, 2024), and impaired academic motivation and achievement (D. Dou & Shek, 2021).

Therefore, exploring risk and protective factors of adolescent IA and their underlying mechanisms matters. Such research can help inform the development of effective interventions to reduce IA among adolescents.

### 1.1. School Disconnectedness and Internet Addiction

School is the primary setting in which adolescents live and study. As a key environmental factor influencing adolescent mental health, schools play a significant role in fostering their healthy development (Kim et al., 2019). However, challenges such as poor school adjustment, exposure to school bullying, and teacher–student conflicts are frequently observed and may contribute to adolescent school disconnectedness (Popp & Peguero, 2012). According to Yu et al. (2011), school disconnectedness reflects a weaker belonging to school and lower support from teachers and classmates.

Stage–environment fit theory suggests that adolescents who feel school disconnectedness may seek alternative ways to compensate for their social needs (Eccles et al., 1993). In contemporary society, the internet serves as an important venue for this purpose (S. Jiang et al., 2025). Online anonymity allows adolescents to temporarily conceal their true identities and interact on platforms such as games and chat software. Existing research supports the stage–environment fit theory, indicating that dissatisfaction with the school climate drives adolescents to fulfill social needs online (Hong et al., 2025; Yuan et al., 2025). Furthermore, adolescents experiencing school disconnectedness are less likely to adhere to school rules (Katsantonis, 2025) and demonstrate less motivation to restrain norm-breaking behaviors (Ivaniushina & Alexandrov, 2022). Thus, adolescents with heightened school disconnectedness are not only motivated to seek alternative social fulfillment online but also lack internalized constraints to regulate excessive internet use. Over time, this pattern may escalate into IA.

Empirical research has supported that school disconnectedness may be a risk factor for adolescent IA (Badenes-Ribera et al., 2019; Peng et al., 2019; P. Wang et al., 2023). Specifically, Peng et al. (2019) found that adolescents with school disconnectedness are more likely to develop IA. Similarly, Badenes-Ribera et al. (2019) and P. Wang et al. (2023) found that school disconnectedness significantly predicts higher susceptibility to social media addiction and online gaming disorder, respectively.

### 1.2. The Mediating Role of Relative Deprivation

Although empirical research has shown that school disconnectedness is a risk factor for adolescent IA, the underlying mechanisms, especially those cognitive pathways such as relative deprivation, remain insufficiently explored.

Relative deprivation refers to an individual’s subjective experience of perceiving that she/he is in a disadvantaged situation by making an upward social comparison with a reference target object and then generating negative emotions such as dissatisfaction and anger (Smith et al., 2012). The cognitive-behavioral model of IA suggests that environmental stressors such as school disconnectedness may increase IA risk through cognitive factors such as relative deprivation (Davis, 2001). Several studies support this theoretical pathway.

First, adolescents who experience school disconnectedness are more likely to develop relative deprivation. Adolescence represents a critical period for self-identity development, during which adolescents frequently engage in social comparisons with peers to construct a stable self-concept (Servidio et al., 2021). Compared to peers with lower school disconnectedness, adolescents with stronger school disconnectedness may perceive themselves at a social disadvantage, leading to relative deprivation in terms of relational and emotional fulfillment (Xiong & Ye, 2016). Moreover, empirical research has shown that both school and social disconnectedness positively predict relative deprivation in adolescents (Y. Chen et al., 2023; F. Dou et al., 2024).

Second, relative deprivation may predict IA. Relative deprivation can trigger a psychological compensation mechanism (Kardefelt-Winther, 2014). As relative deprivation stemming from school disconnectedness intensifies, the internet, with its perceived anonymity and escapism, provides an easily accessible environment for psychological compensation, temporarily easing perceived social deficits. However, due to adolescent still-developing self-control capacity, they become especially vulnerable to the engaging and immersive online activities, which may eventually lead to IA (Tokunaga, 2015). Empirical research supports the link between relative deprivation to IA. For instance, relative deprivation has been found to positively predict online gaming addiction and online social media addiction (Ding et al., 2018; Q. Liu et al., 2021). Additionally, Guo et al. (2024) conducted a longitudinal study and found that relative deprivation predicts later IA over six months.

Although few studies have directly examined the mediating role of relative deprivation between school disconnectedness and adolescent IA, some provide indirect support. For example, J. Li et al. (2025) explored the relationship between school rejection and adolescent IA through a longitudinal study, and found that school rejection at T1 positively predicted relative deprivation at T2, which in turn positively predicted online gaming addiction at T3.

In summary, based on the theoretical and empirical evidence, we hypothesized that relative deprivation may mediate the relationship between school disconnectedness and adolescent IA.

### 1.3. The Moderating Role of Adversity Beliefs

Although school disconnectedness predicts adolescent IA through relative deprivation, this relationship may not be equally strong for all adolescents. The stress-buffering model proposes that positive psychological resources can buffer the negative impact of stressors such as social disconnectedness or relative deprivation on maladaptive outcomes (Cohen & Wills, 1985). Adversity beliefs represent one such potential protective factor (Shek et al., 2003).

Adversity beliefs refer to an individual’s understanding of the nature of adversity, including the causes of, consequences of, and reasonable coping behaviors for adversity, such as “Those who can endure adversity can become stronger than others.” (Shek, 2005). During middle school, pressures stemming from peer relationships, teacher–student interactions, and academic demands intensify, substantially increasing adolescent exposure to adversities. While these challenges are heightened, adolescents ongoing cognitive and psychological development allows them to gradually construct more mature understandings of adversity (Q. Li et al., 2021). Furthermore, within Chinese cultural contexts, stories and values emphasizing resilience and growth through adversity (e.g., the importance of perseverance and learning from hardship) are often transmitted in families and schools during adolescence. Therefore, this period becomes formative for establishing adversity beliefs (B. Yang et al., 2020).

Guided by the stress-buffering model, adversity beliefs may buffer the pathway from school disconnectedness to IA in two ways.

First, adversity beliefs may directly buffer the relationship between school disconnectedness and IA. Empirical research supports this buffering effect. L. Li and Zhu (2022) found that adversity beliefs weakened the link between peer disconnectedness and online gaming addiction. Similarly, M. Zhang et al. (2022) demonstrated that adolescent adversity beliefs mitigate the negative impact of school disconnectedness on gaming addiction. These findings suggest that adversity beliefs can reduce the likelihood that school disconnectedness leads to IA.

Second, adversity beliefs may buffer the relationship between relative deprivation and IA. When experiencing relative deprivation, adolescents with stronger adversity beliefs may be more inclined to employ adaptive coping strategies rather than seeking escapist compensation online. Empirical research indicates that adversity beliefs can mitigate the effects of negative cognition and emotion on online gaming addiction (X. Yang et al., 2022), and help adolescents regulate relative deprivation to avoid gaming addiction (B. Yang et al., 2021). These studies suggest that adversity beliefs may indirectly moderate the relationship between school disconnectedness and IA by alleviating the effect of relative deprivation.

In summary, based on the stress-buffering model and related empirical evidence, we hypothesized that adversity beliefs weaken the positive associations (a) between school disconnectedness and IA, and (b) between relative deprivation and IA.

### 1.4. The Current Study

**Figure 1:**
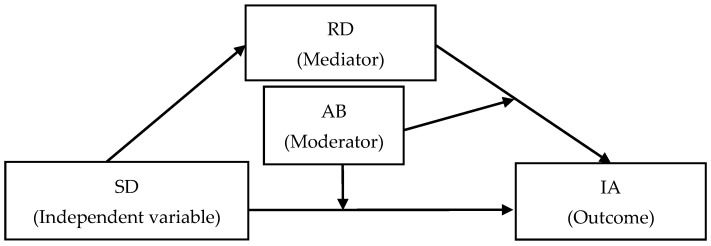
Proposed model of the relationship between school disconnectedness and adolescent IA. SD = School Disconnectedness, RD = Relative Deprivation, AB = Adversity Beliefs, IA = Internet Addiction.

The current study proposes a moderated mediation model linking school disconnectedness to adolescent IA. Based on the stage–environment fit theory, school disconnectedness is expected to be associated with higher adolescent IA. Drawing on the cognitive-behavioral model, relative deprivation, as a cognitive factor, is hypothesized to mediate this relationship. Furthermore, informed by the stress-buffering model, adversity beliefs, as a protective factor, are expected to moderate both the relationship between school disconnectedness and IA and the relationship between relative deprivation and IA. The hypothesized model is presented in Figure 1, and the specific research hypotheses are as follows:

**H1.**  *School disconnectedness will positively predict adolescent IA.*

**H2.**  *Relative deprivation will mediate the relationship between school disconnectedness and adolescent IA. Specifically, school disconnectedness will positively predict relative deprivation, and relative deprivation will positively predict adolescent IA.*

**H3.**  *Adversity beliefs will moderate the relationship between school disconnectedness and adolescent IA. Among adolescents with high adversity beliefs, this relationship will be weaker.*

**H4.**  *Adversity beliefs will moderate the relationship between relative deprivation and adolescent IA. Among adolescents with high adversity beliefs, this relationship will be weaker.*

The proposed framework provides a more nuanced explanation than single mediation or moderation models by integrating environmental, cognitive, and protective factors to explain the link between school disconnectedness and adolescent IA. By identifying relative deprivation and adversity beliefs as key mechanisms, it offers novel theoretical insights and practical implications for prevention and intervention.

## 2. Methods

### 2.1. Participants

Using convenience sampling, this study collected data from three ordinary urban public junior high schools in Yancheng City and Zhenjiang City, Jiangsu Province, China. Yancheng (ranked 89th in GDP nationally) and Zhenjiang (ranked 57th) are midsize cities within the province. The schools covered Grades 7–9, with 10 to 14 classes per grade and approximately 40 students per class, and a balanced gender ratio.

All three schools, which had collaborative relationships with the research team, granted permission to administer the questionnaires during regular class sessions. Sampling was conducted at the class level, with entire classes participating. The homeroom teacher for each class distributed a blank informed consent form via a parents’ WeChat group. The form explained the study’s purpose and content to parents and emphasized the voluntary, anonymous, and confidential nature of participation. Parents provided consent (or refusal) by replying within the group (yielding a high participation rate of 85–90% per class). A participant list was then compiled based on these responses.

Questionnaires and informed consent forms were then distributed in class based on the participant list. All listed students agreed to participate and no withdrawals occurred.

The participants in the current study were aged 12–15 years old and enrolled in Grades 7 and 8. This age range represents a period of heightened vulnerability to IA, resulting from the asynchronous development of the brain’s social-emotional and self-control systems (Pan et al., 2026).

A total of 2591 questionnaires were collected. After excluding 106 questionnaires due to missing data, the final sample comprised 2485 participants (valid response rate = 95.52%). The sample included 1263 boys (*M*_age_ = 13.28 ± 0.76) and 1222 girls (*M*_age_ = 13.25 ± 0.72). Among boys, 572 (45.29%) had migrant status, 387 (30.64%) had left-behind status, 684 (54.16%) were in 7th grade, and 579 (45.84%) were in 8th grade. Among girls, 550 (45.01%) had migrant status, 350 (28.64%) had left-behind status, 679 (55.56%) were in 7th grade, and 543 (44.43%) were in 8th grade.

An a priori power analysis conducted using G*Power 3.1 indicated that a sample size of approximately 263 participants would be sufficient to detect a moderate effect size (*f*^2^ = 0.05) in a multiple regression model with five predictors at an alpha level of 0.05 with 80% statistical power. Given our final sample size of 2485 participants, the study was adequately powered to detect moderate effects.

### 2.2. Measurements

School disconnectedness. It was assessed using the School Connectedness Scale (SCS), developed by Yu et al. (2011). Items were rated on a 5-point Likert scale (1 = *very disagree* to 5 = *very agree*; e.g., “I am proud to belong to this school”). Some items were scored in reverse, with higher scores indicating a stronger school disconnectedness. X. Liu and Liu (2025) reported good applicability among Chinese adolescents (χ^2^/df = 4.51, CFI = 0.90, TLI = 0.88, SRMR = 0.06, RMSEA = 0.04) and a Cronbach’s α of 0.75. In the current study, confirmatory factor analysis (CFA) yielded the following model fit indices: χ^2^/df = 21.07, CFI = 0.95, TLI = 0.93, SRMR = 0.04, RMSEA = 0.09, RMSEA 95% CI [0.084, 0.096], and Cronbach’s α was 0.89.

Relative deprivation. It was measured with the Relative Deprivation Scale (RDS), developed by Ma (2012). Items were rated on a 6-point Likert scale (1 = *very disagree* to 6 = *very agree*; e.g., “I always feel that others have taken what should belong to me”). Higher scores on the scale indicating a stronger relative deprivation. Xuan et al. (2021) reported good applicability among Chinese adolescents (χ^2^/df = 0.52, CFI = 0.96, TLI = 0.99, RMSEA < 0.01) and a Cronbach’s α of 0.62. In the current study, CFA yielded the following model fit indices: χ^2^/df = 2.54, CFI = 0.99, TLI = 0.99, SRMR = 0.01, RMSEA = 0.03, RMSEA 95% CI [0.001, 0.053], and Cronbach’s α was 0.66.

Adversity beliefs. It was assessed using the Chinese Adversity Belief Scale (CABS), developed by Shek (2005). Items were rated on a 6-point Likert scale (1 = *very disagree* to 6 = *very agree*; e.g., “Those who can endure adversity can become stronger than others”). Partial items were scored in reverse, with higher scores indicating greater adversity beliefs. X. Chen et al. (2022) reported acceptable reliability among Chinese adolescents (Cronbach’s α = 0.65). In the current study, CFA yielded the following model fit indices: χ^2^/df = 27.69, CFI = 0.89, TLI = 0.85, SRMR = 0.09, RMSEA = 0.10, RMSEA 95% CI [0.097, 0.110], and Cronbach’s α was 0.69.

Internet addiction. It was measured with the 10-item Internet Addiction Questionnaire (IAQ), revised by Shek et al. (2019). Participants responded “yes” (1 point) or “no” (0 points) to indicate whether they experienced 10 addiction behaviors related to internet use, such as “Do you feel a need to spend more and more time online to achieve satisfaction?”. The total score (0–10), calculated as the sum of “yes” responses, was treated as a continuous variable, consistent with prior research (Zhu et al., 2023). Shek et al. (2019) reported good reliability among Chinese adolescents across three waves (α = 0.79 to 0.82). In the current study, CFA yielded the following model fit indices: χ^2^/df = 5.44, CFI = 0.97, TLI = 0.96, SRMR = 0.02, RMSEA = 0.04, RMSEA 95% CI [0.037, 0.048], and the Cronbach’s α was 0.79.

All items from the measurement scales are presented in Appendix A, Appendix B, Appendix C and Appendix D.

### 2.3. Procedure

The study was approved by the Ethics Committee of the Faculty of Psychology, Tianjin Normal University (protocol number: 2025052618). Prior to data collection, a written informed consent form was obtained from all participants. The informed consent form specified the voluntary nature of participation, guarantees of confidentiality and anonymity, the absence of associated risks, and participants’ right to withdraw from the study at any time without consequences.

With the cooperation of teachers, the questionnaire was distributed to students by the research team during scheduled classes. Participants were instructed to read the instructions carefully before starting. During the approximately 40 min session, they were encouraged to ask the researchers any questions. Upon completion, all questionnaires were collected by the research team. Data collection was conducted between June and July 2025.

### 2.4. Statistical Analyses

First, we performed descriptive statistics and Pearson correlation analyses using SPSS 22.0. Second, to test the hypothesized moderated mediation model, we utilized Hayes’s PROCESS macro (Model 4 and Model 15) for SPSS (Hayes, 2013). Specifically, Model 4 was used to test the mediation effect of relative deprivation on the relationship between school disconnectedness and IA. Model 15 was used to conduct a moderated mediation analysis, testing whether adversity beliefs moderated both the direct effect of school disconnectedness on IA and its indirect effect via relative deprivation. Age, gender, left-behind status, and migrant status were included as control variables.

## 3. Results

### 3.1. Common Method Bias Test

To examine potential common method bias, we performed Harman’s single-factor test via exploratory factor analysis. Results showed that six factors had eigenvalues greater than 1, and the first factor explained 23.02% of the total variance, below the critical threshold of 40% (Malhotra et al., 2017).

For a more rigorous assessment, we applied the unmeasured latent method factor (ULMC) approach using Mplus 7.0 (Tang & Wen, 2020). A CFA incorporating the four trait factors (school disconnectedness, relative deprivation, adversity beliefs, and IA) plus a method factor was compared with the original four-factor model. As shown in Table 1, adding the method factor did not significantly improve model fit (ΔCFI = 0.02, ΔTLI = 0.02, ΔRMSEA = 0.00, ΔSRMR = 0.01). Furthermore, a single-factor model demonstrated poor fit. These results collectively indicate that common method bias is not a serious concern in the current study.

### 3.2. The Correlation Among the Main Variables

As shown in Table 2, school disconnectedness was positively correlated with relative deprivation and IA, and negatively correlated with adversity beliefs. Relative deprivation was negatively correlated with adversity beliefs and positively correlated with IA, while adversity beliefs were negatively correlated with IA.

### 3.3. Testing for the Mediating Role of Relative Deprivation

**Figure 2:**
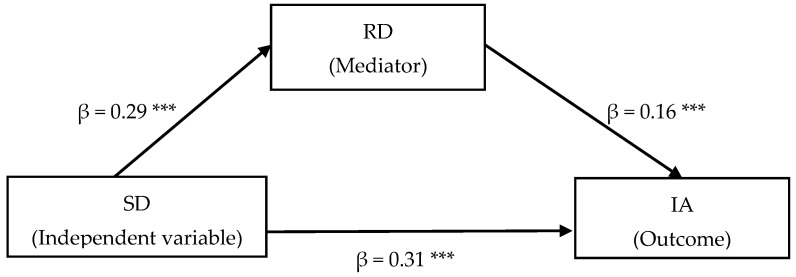
The mediating role of relative deprivation between school disconnectedness and IA. Control variables include gender, age, migrant, and left-behind status. *** *p* < 0.001. SD = School Disconnectedness, RD = Relative Deprivation, AB = Adversity Beliefs, IA = Internet Addiction.

Figure 2 presents the results of the mediation analysis. After controlling for gender, age, migrant status, and left-behind status, school disconnectedness positively predicted IA, β = 0.36, *SE* = 0.02, *t* = 19.11, *p* < 0.001, *R*^2^ = 0.13, *F* = 75.39, *p* < 0.001 (path c); school disconnectedness positively predicted relative deprivation, β = 0.29, *SE* = 0.02, *t* = 15.08, *p* < 0.001, *R*^2^ = 0.09, *F* = 50.28, *p* < 0.001 (path a); and relative deprivation positively predicted IA, β = 0.16, *SE* = 0.02, *t* = 8.40, *p* < 0.001, *R*^2^ = 0.15, *F* = 76.33, *p* < 0.001 (path b). When relative deprivation was added, the direct effect of school disconnectedness on IA remained significant, β = 0.31, *SE* = 0.02, *t* = 16.11, *p* < 0.001 (path c’).

Bootstrapping with 5000 resamples indicated the indirect effect of school disconnectedness on IA through relative deprivation was significant, *ab* = 0.05, *SE* = 0.01, 95% CI [0.03, 0.06]. This indirect effect accounted for 13.89% of the total effect.

### 3.4. Testing for the Moderated Mediation Model

Table 3 presents the results of the moderated mediation model. After controlling for gender, age, migrant status, and left-behind status, the product of school disconnectedness and adversity beliefs negatively predicted IA, β = −0.03, *p* = 0.048, and the product of relative deprivation and adversity beliefs negatively predicted IA, β = −0.10, *p* < 0.001.

**Figure 3:**
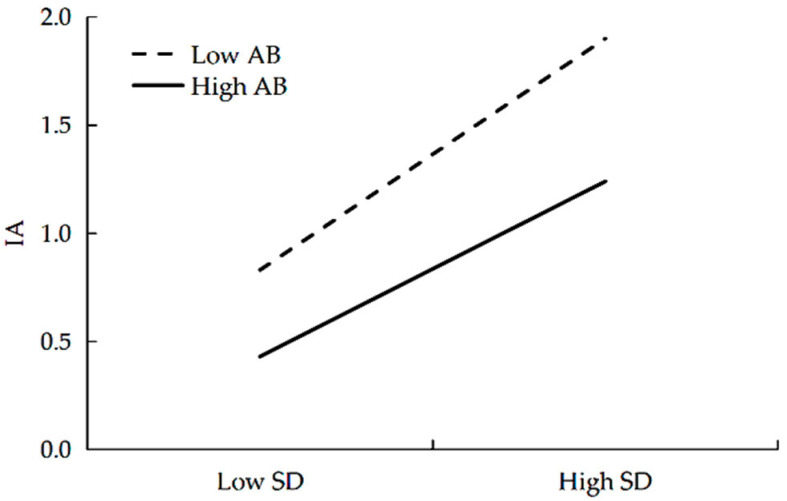
The moderating role of adversity beliefs in the relationship between school disconnectedness and IA. SD = School Disconnectedness, AB = Adversity Beliefs, IA = Internet Addiction.

To interpret these moderating effects of adversity beliefs, simple slope analyses were conducted. As shown in Figure 3, at low levels of adversity beliefs (−1 *SD*), school disconnectedness positively predicted IA, β = 0.58, *SE* = 0.06, *t* = 10.07, *p* < 0.001. At high levels of adversity beliefs (+1 *SD*), school disconnectedness still positively predicted IA, β = 0.43, *SE* = 0.06, *t* = 6.88, *p* < 0.001.

**Figure 4:**
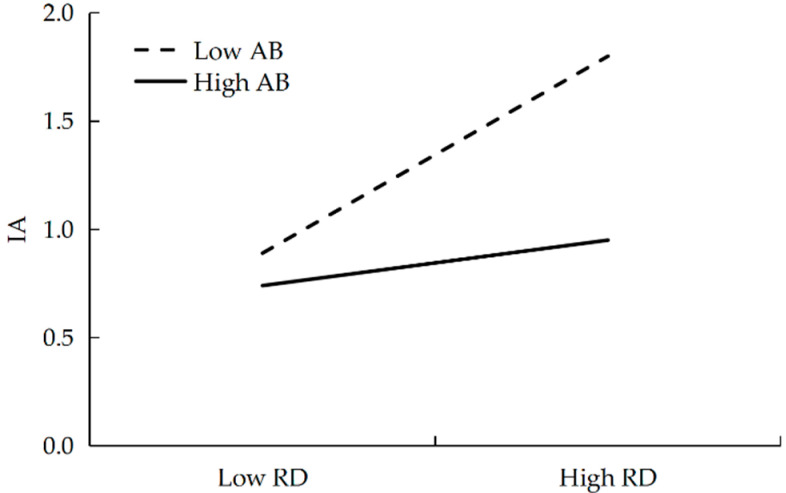
The moderating role of adversity beliefs in the relationship between relative deprivation and IA. RD = Relative Deprivation, AB = Adversity Beliefs, IA = Internet Addiction.

As shown in Figure 4, at low levels of adversity beliefs (−1 *SD*), relative deprivation positively predicted IA, β = 0.46, *SE* = 0.05, *t* = 10.10, *p* < 0.001. At high levels of adversity beliefs (+1 *SD*), relative deprivation still positively predicted IA, β = 0.10, *SE* = 0.05, *t* = 2.17, *p* = 0.030.

The conditional indirect effects were examined, as shown in Table 4. At low levels of adversity beliefs (−1 *SD*), the indirect effect of relative deprivation was significant, *ab* = 0.08, *SE* = 0.01, 95% CI [0.05, 0.10]. At high levels of adversity beliefs (+1 *SD*), the indirect effect of relative deprivation was not significant, *ab* = 0.02, *SE* = 0.01, 95% CI [−0.001, 0.03].

## 4. Discussion

In contemporary society, adolescent IA has become a salient public health issue. However, its underlying risk and protective mechanisms remain insufficiently explored. Drawing on the stage–environment fit theory, school disconnectedness was proposed as a risk factor for adolescent IA (H1). Furthermore, based on the cognitive–behavioral model, relative deprivation was hypothesized to mediate the relationship between school disconnectedness and IA (H2). Finally, drawing on the stress-buffering model, adversity beliefs were expected to moderate both the direct link between school disconnectedness and IA (H3) and the link between relative deprivation and IA (H4).

Therefore, the current study aimed to examine the relationship between school disconnectedness and adolescent IA by testing a moderated mediation model. Specifically, the model incorporated relative deprivation as a mediator and adversity beliefs as a moderator. By integrating these theoretical perspectives, the current study sought to advance understanding of mechanisms underlying school disconnectedness and IA, and offering insights for prevention and intervention.

### 4.1. Main Findings

#### 4.1.1. The Direct Effect of School Disconnectedness on IA

Consistent with H1, school disconnectedness was a significant risk factor for adolescent IA. This finding aligns with prior research. For example, Peng et al. (2019) found that adolescents with higher school disconnectedness were more prone to IA than their peers. It also supports the stage–environment fit theory (Eccles et al., 1993), which emphasizes the importance of contextual support in meeting social needs.

Schools are a primary setting where adolescents fulfill essential social needs such as belonging, support, and acceptance (Tian et al., 2016). When these needs are unmet at school, students may experience social isolation and turn to alternative avenues to compensate for this deficit (Santini et al., 2021).

In this context, the internet, characterized by its accessibility and interactivity, readily serves as a substitute venue for need fulfillment. By 2021, China had 191 million adolescent internet users, representing a 96.8% penetration rate within this group (China Internet Network Information Center, 2021). Such widespread access increases the likelihood that adolescents will use online spaces to escape real-world stressors. Indeed, online engagement can alleviate loneliness and enhance short-term life satisfaction (Zhou & Cheng, 2022). However, due to adolescents still developing self-control, they are particularly vulnerable to the reinforcing properties of the internet. Consequently, such internet use may progress into addiction over time (Liang et al., 2025).

#### 4.1.2. The Mediating Effect of Relative Deprivation

Consistent with H2, relative deprivation was an important explanatory mechanism for the relationship between school disconnectedness and adolescent IA. Adolescents with higher school disconnectedness reported higher relative deprivation, which in turn increased IA. This aligns with Chang et al. (2025), who found that school bullying increased adolescent relative deprivation, thereby elevating their vulnerability to IA. The finding also supports the cognitive-behavioral model of IA, which posits that school disconnectedness, as an environmental stressor, increases IA through internal cognitive factors like relative deprivation (Davis, 2001).

Adolescents seek positive interactions with peers and teachers, such as sharing experiences and collaborative study (Mustari et al., 2022). School disconnectedness limits these social opportunities and relational benefits. This perceived disadvantage can foster a sense of relative deprivation, as adolescents compare their unmet needs with the fulfilling school experiences of peers (T. Jiang & Chen, 2020).

The relative deprivation stemming from school disconnectedness may generate a psychological need to escape this aversive state (F. Dou et al., 2024). The online environment offers an alternative space to temporarily evade real-world pressures and reconstruct self-worth. In an effort to compensate for their perceived social disadvantage, adolescents may increasingly invest time and effort online. This maladaptive coping strategy, combined with the reinforcing nature of the online environment, can lead to a cycle of excessive internet use that ultimately develops into IA (Guo et al., 2024).

However, it is important to note that while school disconnectedness and relative deprivation are significant contributors to adolescent IA, they are not the sole factors. First, school disconnectedness is not the only external risk factor; family-related variables, such as parental neglect (F. Li et al., 2023), harsh parenting (Kong et al., 2026), and family conflict (Sun et al., 2025), also elevate IA. Second, relative deprivation is only one potential pathway linking school disconnectedness to IA. Other internal cognitive mechanisms, such as low self-control (Su et al., 2024) and negative self-expectations (Lin & Zha, 2025), may also increase vulnerability. Thus, school disconnectedness and relative deprivation should be viewed as key, yet non-exclusive, components within a broader etiological framework of adolescent IA.

#### 4.1.3. The Moderating Effect of Adversity Beliefs

First, consistent with H3, the positive association between school disconnectedness and IA was weaker among adolescents with higher adversity beliefs. This finding aligns with prior research (L. Li & Zhu, 2022) and supports the stress-buffering model (Cohen & Wills, 1985). School disconnectedness can be perceived as an adversity, leading to loneliness and stress (Santini et al., 2021). In such circumstances, adolescents with stronger adversity beliefs are more likely to maintain a positive outlook, perceive themselves as active agents, and seek meaning in challenges (Shek, 2005). Rather than escaping online, they may reframe adversity as a catalyst for growth, reducing their reliance on maladaptive coping strategies like excessive internet use.

Second, consistent with H4, adversity beliefs also buffered the relationship between relative deprivation and IA. This suggests that adversity beliefs may enhance adolescents’ capacity to regulate emotions and cognitions when facing relative deprivation. Prior research indicates that individuals with higher adversity beliefs show an attentional bias toward positive stimuli, reflecting a more adaptive coping style (Gao et al., 2025). Similarly, adversity beliefs can buffer the impact of negative emotions and cognitions on IA (X. Yang et al., 2022). In essence, adolescents with stronger adversity beliefs tend to be more optimistic, viewing adversity as an opportunity for development (Shek, 2005). This may help them manage emotions and cognitions more effectively, reducing their reliance on the internet (L. Yang et al., 2017).

However, the current study also acknowledges that the buffering effect of adversity beliefs was limited. Even at high levels of adversity beliefs, school disconnectedness and relative deprivation remained significant positive predictors of IA. This indicates that while adversity beliefs can mitigate the association of school disconnectedness or relative deprivation with IA, they cannot completely offset these relationships. Therefore, researchers should maintain an objective perspective on their protective role and avoid overestimation.

### 4.2. Theoretical and Practical Implications

#### 4.2.1. Theoretical Implication

While the stage–environment fit theory and the cognitive-behavioral model posit a link between school disconnectedness, relative deprivation, and IA, they tend to overlook potential protective factors. Based on the stress-buffering model, the current study proposed that adversity beliefs can buffer both the direct link between school disconnectedness and IA and the mediating pathway through relative deprivation. In doing so, the current study enriches an environment–cognition–behavior model through the incorporation of a buffering mechanism. This integrated model not only accounts for risk but also explicitly incorporates a protective component, thereby offering a more nuanced and balanced theoretical framework for understanding adolescent IA.

#### 4.2.2. Practical Implication

The current study identified school disconnectedness as a significant risk factor for adolescent IA. Therefore, interventions aimed at enhancing school connectedness appear promising. According to existing research, middle schools can implement mental health programs that include role-playing, interactive workshops, and team competitions to foster belonging among students (Barzilay et al., 2025). Alternatively, after-school group art programs can help adolescents experience the joy of friendship and growth within the school environment (Lee et al., 2025). Strengthening school connectedness through such programs may help reduce adolescent IA.

Furthermore, cultivating adversity beliefs may also mitigate IA. Middle schools can implement “adversity beliefs education” programs in which teachers guide adolescents to recognize and understand adversity, reappraise adversity, promote peer collaboration, and help them collectively overcome challenges, thereby fostering a belief in their ability to overcome adversity (M. Wang et al., 2024).

Overall, researchers should collaborate with middle schools to design and implement intervention programs that enhance school connectedness and adversity beliefs, addressing adolescent IA.

### 4.3. Limitations and Future Directions

The current study has several limitations.

First, the current study used a cross-sectional design, which limits causal inferences regarding the relationship between school disconnectedness and IA. Future studies could further employ longitudinal, qualitative or mixed-methods designs to explore this relationship.

Second, while researchers frequently employ the RDS and CABS, the internal consistency reliability of these measures is only moderate (X. Chen et al., 2022; Xuan et al., 2021). Although coefficients of internal consistency in the range of 0.65–0.80 are generally acceptable (Vaske et al., 2016), moderate reliability could attenuate observed effect sizes, meaning the significant associations found here are likely conservative estimates of the true relationships (Henson, 2001). Future studies are encouraged to improve these measures.

Third, the current findings rely on adolescent self-reports, lacking evaluations from teachers or parents. This limitation was driven by practical constraints: large class sizes precluded accurate teacher assessments of all students, and some parents were unavailable due to working in other regions. Future work could enhance validity by incorporating multi-informant reports (e.g., from parents or teachers) or objective behavioral data.

Fourth, given that adversity beliefs provide only partial protection, future research should explore complementary protective factors, such as out-of-school social support (J. Liu & Wang, 2018), to identify optimal intervention combinations.

Fifth, the generalizability of the findings may be limited as the sample was drawn only from Jiangsu Province, China. Future studies could recruit participants from diverse regions across China to examine potential urban–rural or regional differences. Moreover, while grounded in Chinese culture, the cultivation of adversity beliefs is not culturally exclusive (Jobson et al., 2022). Given the global concern about IA (X. Liu et al., 2025), cross-cultural comparisons of the prevalence and protective role of adversity beliefs would be valuable.

Finally, the current study focused primarily on the relationship between school disconnectedness and adolescent IA. Adolescents develop within interconnected ecological systems, and various external (e.g., family, societal) and internal (e.g., cognitive, emotional) factors also influence IA (Y. Zhang et al., 2025). Future research could incorporate additional external and internal factors to test more comprehensive theoretical models.

## 5. Conclusions

The current study examined the relationship between school disconnectedness and adolescent IA. The findings reveal that school disconnectedness is a risk factor for adolescent IA. Furthermore, it increases adolescent IA by enhancing relative deprivation. In addition, adversity beliefs play a protective role. Adolescents with high adversity beliefs are less likely to develop IA even if they experience school disconnectedness and relative deprivation.

## Figures and Tables

**Table 1:** Model fit indices for confirmatory factor analysis of different models.

Model	χ^2^	*df*	TLI	CFI	RMSEA	SRMR
Five factors	3151.44	458	0.89	0.90	0.05	0.05
Four factors	3730.94	489	0.87	0.88	0.05	0.06
Single factor	14,181.58	497	0.47	0.50	0.11	0.16

**Table 2:** Descriptive statistics and correlations between the main variables.

Variables	*M* (*SD*)	1	2	3	4	5	6	7
1 Gender	0.51 (0.50)	1						
2 Age	13.26 (0.74)	0.02	1					
3 Migrant	0.45 (0.50)	0.003	0.004	1				
4 Left-behind	0.30 (0.46)	0.02	0.03	0.05 *	1			
5 SD	2.23 (0.86)	−0.03	0.07 **	−0.001	0.04 *	1		
6 RD	2.60 (1.07)	0.08 ***	−0.02	0.03	0.04 *	0.29 ***	1	
7 AB	4.44 (0.80)	0.07 ***	−0.08 ***	−0.05 *	−0.02 *	−0.49 ***	−0.17 ***	1
8 IA	1.19 (1.86)	0.04 *	−0.01	0.04 *	0.03	0.36 ***	0.26 ***	−0.29 ***

*n* = 2485, * *p* < 0.05, ** *p* < 0.01, *** *p* < 0.001. SD = School Disconnectedness, RD = Relative Deprivation, AB = Adversity Beliefs, IA = Internet Addiction. Gender was coded as 0 and 1, 0 = girl, 1 = boy; Migrant was coded as 0 and 1, 0 = no, 1 = yes; Left-behind was coded as 0 and 1, 0 = no, 1 = yes.

**Table 3:** Testing the moderated mediation model between school disconnectedness and IA.

Predictor	RD			IA		
	β	*SE*	*t*	β	*SE*	*t*
Gender	0.08	0.02	4.36 ***	0.04	0.02	2.37 *
Age	−0.04	0.02	2.15 *	−0.04	0.02	2.08 *
Migrant	0.03	0.02	1.64	0.03	0.02	1.84
Left-behind	0.03	0.02	1.51	0.01	0.02	0.30
SD	0.29	0.02	15.08 ***	0.23	0.02	10.77 ***
AB				−0.15	0.02	7.02 ***
SD × AB				−0.03	0.02	1.98 *
RD				0.16	0.02	8.41 ***
RD × AB				−0.10	0.02	5.78 ***
*R* ^2^	0.09			0.19		
*F*	50.28 ***			63.59 ***		

* *p* < 0.05; *** *p* < 0.001. SD = School Disconnectedness, AB = Adversity Beliefs, RD = Relative Deprivation, IA = Internet Addiction. Gender was coded as 0 and 1, 0 = girl, 1 = boy; Migrant was coded as 0 and 1, 0 = no, 1 = yes; Left-behind was coded as 0 and 1, 0 = no, 1 = yes.

**Table 4:** Conditional indirect effects of relative deprivation between school disconnectedness and IA.

		Conditional Indirect Effect			
Mediator	Moderator	Indirect Effect	*SE*	95% Confidence Interval	
				LLCI	ULCI
Relative Deprivation	Low	0.08	0.01	0.05	0.10
	Medium	0.05	0.01	0.03	0.06
	High	0.02	0.01	−0.001	0.03

LLCI = Lower Limit Confidence Interval, ULCI = Upper Limit Confidence Interval.

## Data Availability

The raw data supporting the conclusions of this article will be made available by the authors on request.

## Appendix A. School Connectedness Scale (SCS)

A1. I can rely on my classmates when I encounter difficulties.
A2. I don’t like the teachers at my school.
A3. I feel happy and safe at school.
A4. My classmates can really help me.
A5. The teachers are very caring and supportive.
A6. I am glad to be a part of this school.
A7. My classmates can share joys and sorrows with me.
A8. The teachers at our school treat students fairly.
A9. I am proud to belong to this school.
A10. I find it difficult to get along with my classmates.

## Appendix B. Relative Deprivation Scale (RDS)

A1. Considering the effort I have put in, my life should be better than it is now.
A2. I always feel that others have taken what should belong to me.
A3. Compared to people around me, I get the short end of the stick in various aspects of life and studies.
A4. Most wealthy people in society have become rich through dishonorable means.

## Appendix C. Chinese Adversity Belief Scale (CABS)

A1. Those who can endure adversity can become stronger than others.
A2. One’s quality of life is determined by fate.
A3. A person with strong determination will ultimately overcome adversity.
A4. With enough confidence, one is bound to succeed.
A5. Poverty stifles ambition.
A6. No pain, no gain.
A7. Human will can ultimately overcome the unfairness of fate.
A8. Focusing on what you have is the key to enduring joy.
A9. One can only become strong through continuous effort and self-improvement.

## Appendix D. Internet Addiction Questionnaire (IAQ)

A1. Do you feel preoccupied with the Internet or online services and think about it while offline?
A2. Do you feel a need to spend more and more time online to achieve satisfaction?
A3. Have you repeatedly made unsuccessful efforts to control, cut back, or stop internet use?
A4. Do you feel restless, moody, depressed, or irritable when attempting to cut down or stop internet use?
A5. Do you use the internet as a way of escaping from problems or of relieving a dysphoric mood (e.g., feelings of helplessness, guilt, anxiety, depression)?
A6. Have you concealed the extent of your internet use from your parents or teachers?
A7. Have you jeopardized or risked the loss of a significant friendship or studies because of the internet?
A8. Do you stay online longer than originally intended?
A9. Do you keep returning even after spending too much money on online fees?
A10. Do you feel depressed, irritable, moody, or anxious when you are offline?
